# Pedal effect: aerobic and facultative anaerobic *Enterobacter hormaechei* maintain the homeostasis of the house fly gut microbiota under different oxygen conditions

**DOI:** 10.1186/s13071-025-07013-5

**Published:** 2025-12-06

**Authors:** Kexin Zhang, Shumin Wang, Ying Li, Xinrui Zhang, Yansong Yin, Xinxin Kong, Wenjuan Liu, Dawei Yao, Ruiling Zhang, Zhong Zhang

**Affiliations:** 1 Dermatology Hospital of Shandong First Medical University, Jinan, China; 2 Shandong Provincial Institute of Dermatology and Venereology, Shandong Academy of Medical Sciences, Jinan, China; 3 School of Life Science, Shandong First Medical University (Shandong Academy of Medical Sciences), Taian, China; 4 Medical College of NanKai University, Tianjin, 300071 China; 5 School of Clinical Medicine, Shandong Second Medical University, Weifang, China; 6 Collaborative Innovation Center for the Origin and Control of Emerging Infectious Diseases, Shandong First Medical University (Shandong Academy of Medical Sciences), Taian, China; 7 School of Clinical and Basic Medical Science, Shandong First Medical University and Shandong Academy of Medical Sciences, Jinan, China; 8 Department of Laboratory Medicine, Sun Yat-Sen Memorial Hospital, Sun Yat-Sen University, Shanwei, China; 9 Shandong Institute of Endocrine and Metabolic Diseases, Shandong First Medical University, Jinan, Shandong China; 10 School of Life Science, Shandong Second Medical University, Weifang, China; 11 Medical Science and Technology Innovation Center, The First Affiliated Hospital of Shandong First Medical University, Jinan, China

**Keywords:** Intestinal flora, House fly, Aerobic bacteria, Facultative anaerobic bacteria, *E. hormaechei*, Phage

## Abstract

**Background:**

House fly gut microbiota plays a major role in regulating larval development and immune responses. As the house fly gut microbiota is complex with different functions, the role of many gut microbiota in the host remains unknown. The differential oxygen content in the midgut and hindgut of house fly intestines creates distinct gut microenvironments, resulting in variations in the composition of gut microbiota. Research on the distribution of aerobic and facultative anaerobic bacteria in different parts of the intestine—and how they interact with each other—is helpful for exploring the interaction between intestinal bacteria and the host in aerobic and anaerobic states. However, there have been no mechanistic studies on the interactions of gut bacteria in different oxygen microenvironments within the house fly.

**Methods:**

In this study, aerobic *Enterobacter hormaechei* EhX and facultative anaerobic *E. hormaechei* EhY were isolated from the guts of house fly larvae. The specific phages, EhXP and EhYP, were isolated against these two bacteria, with phage EhXP and EhYP specifically targeting EhX and EhY, respectively. Furthermore, the role of the aerobic–anaerobic interaction in *E. hormaechei* during larval development was investigated using phage and bacterial feeding experiments.

**Results:**

The results showed that the addition of aerobic *E. hormaechei* EhX or facultative anaerobic *E. hormaechei* EhY alone to the diet promoted larval growth and phenoloxidase activity. However, in the phage-treated groups, the opposite effect was observed. In contrast, EhY was the dominant bacteria in larval hindgut. Conversely, EhX was the main bacteria in the midgut of larvae. Aerobic/facultative anaerobic gut bacteria competed and cooperated to maintain a stable composition of larval gut microbiota, forming a negative correlational “pedal effect” promoting larval development. Through high-throughput sequencing it was demonstrated that the abundance of beneficial bacteria increased in the bacteria treatment group. The number of harmful bacteria increased in the phage treatment group.

**Conclusions:**

The results revealed the crucial role of aerobic and facultative anaerobic *E. hormaechei* in the house fly. These results highlight the potential of future applications of multiple phages for the study of insect intestinal flora.

**Graphical Abstract:**

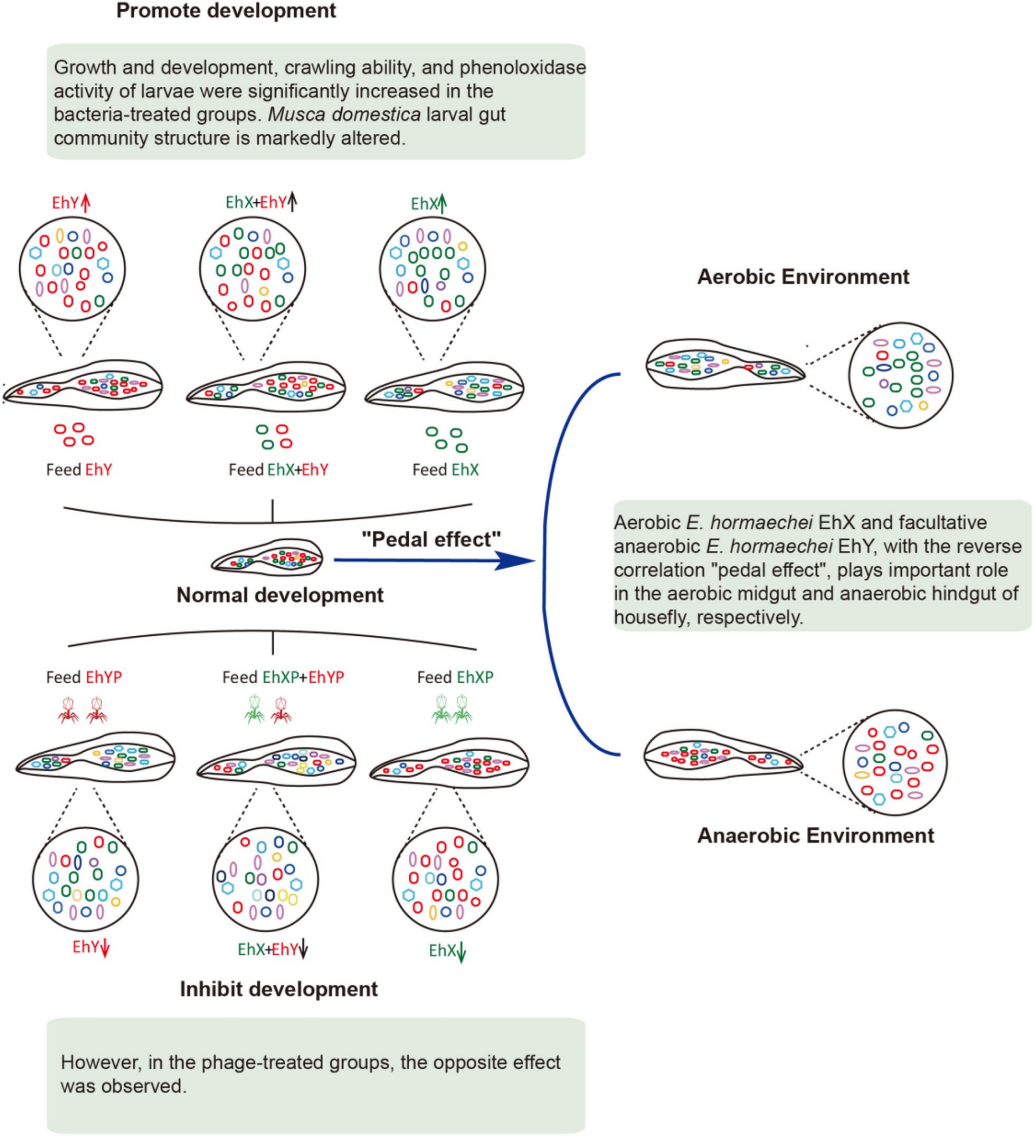

**Supplementary Information:**

The online version contains supplementary material available at 10.1186/s13071-025-07013-5.

## Background

Insect guts provide appropriate environments for microbial colonization. The gut microbiome participates in the life activities of most insects and performs critical functions in the physiology of the host, particularly in the modulation of the host’s immune response and the outcome of pathogen infection [1–4]. Studies have shown that the insect gut microbiome can aid in the host’s digestion of food and complex organics (such as flavonoids and outer pollen wall components [5]), improve host immunity against pesticide damage [6], affect host life [7], and help in providing resistance against pathogenic fungi [8]. The gut microbiome plays a crucial role in various insect species by aiding in detoxification and digestion, providing essential nutrients, enhancing reproductive success, and influencing host–pathogen interactions [9–15]. These findings suggest the potential impact of gut bacteria on insect hosts is beneficial in shaping the composition of the normal gut microbiota. Understanding the interactions between the insect and gut microbiota may provide new insights into pathogen–insect interactions and may assist in the development of new insect control strategies.

The basic structure of the insect digestive tract is similar between species, although it exhibits variations associated with adaptations to different feeding modes. The gut has three main regions: the foregut, the midgut (or ventriculus), and the hindgut [3], and serves as an ideal and nutrient-rich ecological niche, supporting the proliferation of various microbial communities [16]. The insect digestive tract exhibits significant morphological and physicochemical variations, which greatly influence the structure of the microbial community [3]. To date, there is limited literature on how the gut microbiota varies depending on diet and gut compartment. Oxygen content can influence community composition, and low oxygen promotes the domination of anaerobic bacteria [17, 18]. Insects exhibit distinct bacterial communities in the midgut and hindgut, with the midgut typically hosting aerobic bacteria, and the hindgut being dominated by anaerobic microorganisms. While aerobic bacteria have been extensively studied, the roles and interactions of facultative anaerobic bacteria across different gut regions remain underexplored [16, 19–21]. However, interactions between other aerobic and facultative anaerobic bacteria in the gut of house fly larvae and their impact on the host require further investigation. The investigation of the interaction between aerobic and facultative anaerobic bacteria in the gut can help to reveal the interactions between these bacteria and hosts in the aerobic and anaerobic states. However, extensive research mainly focuses on the influence of specific bacteria or their overabundance on insect development [22, 23]. The effects of the absence of certain bacteria on insect health and gut microbiota need to be further explored. Research on the insect gut microbiome is mostly based on the mode of “total removal and replenishment” [8], which perturbs nontargeted bacteria in the gut, causing broad and potentially long-lasting perturbations. Therefore, approaches are needed to more precisely and rationally modulate gut bacteria to remodel the gut microbiota within a complex community.

Bacteriophages are self-replicating viruses that specifically infect and parasitize bacteria through interactions with host bacterial receptors [24]. Owing to their specificity, phage therapy is considered suitable for targeting and removing specific bacteria [25–27]. As specific bacterial predators, phages can change microbial diversity through Red Queen/kill-the-winner dynamics [28, 29]. While phage predation directly targets susceptible bacteria, it also triggers cascading effects on nontargeted bacteria through interbacterial interactions, leading to changes in species abundance [30]. Studies have highlighted the role of phages in regulating insect gut bacterial communities [31–34] and their therapeutic potential in animals [30, 32, 35–38] and plants [39, 40]. Phages are highly host-specific and propagate rapidly in the presence of host bacteria, minimally affecting nontarget bacteria, making them promising tools for precise targeting [41, 42]. The “beneficial bacteria” in house fly larvae can promote larval development and humoral immunity, while the “harmful bacteria” are the opposite [22]. The gut bacteria knockdown technique using bacteriophages by Zhang et al. was used to study the function of house fly larvae gut bacteria [33, 43].

In our previous research, we established that phages cause targeted destruction of susceptible *Pseudomonas aeruginosa* in the guts of house fly larvae, and preliminarily explored the influence of *P. aeruginosa* on larval development and the perturbation of the gut microbiome [43]. There is a lack of research on the effects of combining two phages on the gut microbiome of house flies and the roles of aerobic and facultative anaerobic bacteria, so our study investigated these interactions using single and combined phage treatments to better understand their impact on insect health. In this study, two strains of *E. hormaechei* were isolated from the guts of house fly larvae. *E. hormaechei* EhX and *E. hormaechei* EhY were isolated under aerobic and anaerobic conditions, respectively. The EhYP phage of *E. hormaechei* EhY was isolated from sewage. The phage EhXP was isolated from larval house fly gut in our previous research [33]. Phages have high specificity for their host bacteria and thus, phage EhXP only infects the susceptible bacteria *E. hormaechei* EhX, but not *E. hormaechei* EhY. Phage EhYP infects only the targeted bacteria *E. hormaechei* EhY, but not *E. hormaechei* EhX. On this basis, we added *E. hormaechei* EhX/EhY or phage EhXP/EhYP to the larval diet, respectively. Furthermore, we evaluated larval development in different treatment groups, gut bacteria composition, and the function of the two strains of *E. hormaechei*. Our research revealed the major role of aerobic and facultative anaerobic *E. hormaechei* in the house fly aerobic midgut and anaerobic hindgut, constantly maintaining the composition of gut bacteria in house fly larvae through competition and cooperation, forming a negative correlation “pedal effect.”

## Methods

### Animal and microbial strains

A house fly (*Musca domestica*) population was reared and maintained in the Laboratory of Vector and Insect Diseases of Shandong First Medical University since 2005 [43].

*E. hormaechei* EhX and *E. hormaechei* EhY were isolated from the house fly larval gut under aerobic and facultative anaerobic conditions, respectively, using traditional isolation and culture methods and anaerobic techniques (anaerobic airbag and tank), as shown in our previous work [21, 33]. The bacteria were cultivated using nutrient agar (NA) medium plates and LB liquid medium. Isolation was conducted at the Qingdao Sequencing Department, Beijing Ruibo Xingke Biotechnology Co., Ltd., where sequencing was performed. Amplification of the 16S ribosomal DNA (rDNA) utilized primers 27F (AGAGTTTGATCCTGGCTCAG) and 1492R (TACGGCTACCTTGTTACGACTT), with subsequent BLAST analysis in the GenBank database to identify closely related sequences. The sequences amplified by 16S rRNA gene sequencing technology were aligned and analyzed on the NCBI website. Sequences with over 97% homology were used as references.

### The bacterial growth rate determination of *E. hormaechei* EhX and *E. hormaechei* EhY in aerobic and anaerobic states

To demonstrate that the *E. hormaechei* EhX and *E. hormaechei* EhY have two different shapes in aerobic and anaerobic states, bacterial growth tests were carried out in nutrient agar (NA) medium plates under aerobic and anaerobic culture conditions.

The growth rates of *E. hormaechei* EhX and *E. hormaechei* EhY were measured. *E. hormaechei* EhX were inoculated in Luria Bertani (LB) liquid medium and cultured at 37 °C overnight (OD600 = 0.8) under aerobic culture conditions. *E. hormaechei* EhY was treated the same as *E. hormaechei* EhX except for anaerobic state culture. Six 6 mm-diameter sterile filter papers were placed symmetrically on both sides of the agar medium, 5 µL *E. hormaechei* EhX was added to the filter papers. All plates were cultured in aerobic and anaerobic condition for 24, 48, and 72 h at 37 °C. *E. hormaechei* EhY was treated with the same process as *E. hormaechei* EhX. Growth rates of *E. hormaechei* EhX and *E. hormaechei* EhY in the aerobic and anaerobic states were assessed by measuring colony size. Experiments were performed with 12 independent biological replicates.

### Bacteriophage isolation and identification

The phages were isolated from the house fly larval gut and the Tai’an Sewage Treatment Plant as described previously [21, 33]. *E. hormaechei* EhX and *E. hormaechei* EhY isolated from the house fly larval gut under aerobic and anaerobic conditions were used as host bacteria for phage screening. Phage EhXP (Phc, MZ669808) was isolated and purified in our previous research [33]. In this experiment, sewage from the Tai’an treatment plant in Shandong Province was collected for phage isolation. *E. hormaechei* EhY isolated from the intestinal tracts of house fly larvae under facultative anaerobic conditions was used as host bacteria to screen phages. Phage EhYP was purified by a double-layer agar plate method based on our previous research report [43]. The high concentration phage stocks were stored at 4 °C. The purified phage preparation was imaged under transmission electron microscope (H-7700, Hitachi, Japan).

In order to determine the host range [44] of phage EhYP, the spot test was used. The ability of phage KYP to infect different bacteria was tested against 11 cultivable bacteria in the house fly larval gut (Table S1). To do so, 10 µL of the diluted phage EhYP (10^4^ PFU mL^−1^) was dropped onto a double-layer nutrient agar (NA) plate containing the test bacterial strain. After incubation overnight at 37 °C, the presence of a clear zone indicated that the bacteria were sensitive to phages. In our previous study, the host range of phage EhXP was determined [33]. The basic biological characteristics of EhYP were determined according to our previous research [21].

### Experimental design of insect feeding and *E. hormaechei* (EhX and EhY) and phage (EhXP and EhYP)

To study the effects of *E. hormaechei* EhX/EhY on larval development, *E. hormaechei* EhX/EhY were cultured in Luria Bertani (LB) liquid media. To study the effects of a *E. hormaechei* EhX–EhY mixture on larval development, *E. hormaechei* EhX and EhY were mixed 1:1 at the same concentration of 10^9^ CFU mL^−1^.

House fly larvae from different groups that were fed: Luria Bertani (LB) liquid medium, *E. hormaechei* EhX (10^9^ CFU mL^−1^), *E. hormaechei* EhY (10^9^ CFU mL^−1^), *E. hormaechei* EhX (10^9^ CFU mL^−1^) and *E. hormaechei* EhY (10^9^ CFU mL^−1^), phage EhXP (10^9^ PFU mL^−1^), phage EhYP (10^9^ PFU mL^−1^), and phage EhXP (10^9^ PFU mL^−1^) and phage EhYP (10^9^ PFU mL^−1^), were named LB, EhX, EhY, EhXY, EhXP, EhYP, and EhXYP, respectively, based on our previous research [21].

### House fly larvae crawling ability assay

House fly larvae’s crawling ability was evaluated using the protocol described in our previous study [34]. Six larvae from various groups were selected on the third day post-feeding and positioned on a crawling trace medium at room temperature, permitting unrestricted movement on the substrate. Following a 15-min interval, photographic documentation of the larvae’s crawling paths was obtained, and Digitizer^®^ 4 was employed for tracing and calculation purposes.

### Effects of the addition and removal of *E. hormaechei* on phenoloxidase activity in house fly larvae

Larval phenoloxidase activity was determined using the method described in our previous research [22, 34]. The feeding trial involving house fly larvae spanned 4 days, with daily collection of samples from each group. Each house fly larva sample was homogenized in a centrifuge tube filled with 0.5 mol/L phosphate buffer (pH = 7.0). After centrifugation at 4 °C and 12,000 rpm for 20 min, the supernatant was extracted. The enzymatic reaction setup was identical to the methodology previously outlined. Following a 15-min incubation in a 25 °C water bath, the optical density at 405 nm (OD_405_) was determined.

### Plate confrontation assay between *E. hormaechei* and cultivable bacteria in the house fly larval gut

*E. hormaechei* EhY cultures were inoculated on half of the nutrient agar plate using the spread plate method with a sterile cotton swab, with the opposite side of the agar plate used as a negative control. Subsequently, 10 µL of the isolated cultivable gut bacteria, including *E. hormaechei* EhX, *P. aeruginosa* Y12, *P. verticola* Pv, and *P. stuartii* Ps were added to the filter papers. The plate confrontation assay of EhX was determined according to our previous research [33]. In addition, a plate confrontation assay of EhX against EhY was performed following our previous protocol [21]. The *E. hormaechei* EhX cultures were inoculated on half of the nutrient agar plate using the spread plate method with a sterile cotton swab, and the opposite side of the agar plate was used as a negative control. Two 6 mm-diameter sterile filter papers were placed symmetrically on both sides of the agar medium. Subsequently, 10 µL of the isolated cultivable gut bacteria *E. hormaechei* EhY was added to each filter paper.

### Sequencing and bioinformatics analysis

Whole-genome sequencing of phage EhYP, 16S rRNA gene high-throughput sequencing of house fly gut microbiota, and bioinformatics analysis were carried out using the same method as described in our previous research [43].

Phage genomes were sequenced utilizing the Illumina HiSeq 4000 system (Illumina Inc., San Diego, CA, USA) and subsequently assembled de novo with the MetaviralSPAdes tool. The phage sequences referenced in this study have been deposited in the GeneBank database, accessible under the accession numbers EhXP: Phc, MZ669808 and EhYP: OQ884031.

Intestinal bacterial DNA extraction and subsequent analysis of gut composition alterations via Illumina MiSeq sequencing were executed. MENAP software facilitated the analysis of operational taxonomic unit (OTU) data from each sample, yielding edge and node information. The network was performed using Gephi. The parameters used in drawing mainly include: node size as degrees (the more connections, the higher the degree), the node color as phylum classification, the edge color as positive and negative correlation, and the node layout as the Fruchterman Reingold layout.

### Bacterial abundance assay of *E. hormaechei* EhX/EhY in the midgut and hindgut of house fly larvae

Owing to the high specificity of phages, phage EhXP/EhYP could rapidly amplify only in the presence of their host bacteria. Therefore, the bacterial load of *E. hormaechei* EhX/EhY in the guts of house fly larvae could be analyzed by measuring the titers of their specific phages in vitro. As the midgut and hindgut contain different oxygen conditions, phage titer analysis was incorporated to analyze the dynamic bacterial load of *E. hormaechei* EhX and *E. hormaechei* EhY in midgut and hindgut according to our previous research [21]. The normally reared house fly larvae were cultured to 3-day-old larvae, and the midgut and hindgut were dissected. The host specificity of two phages, EhXP and EhYP, was used to measure the concentrations of *E. hormaechei* EhX and *E. hormaechei* EhY in the midgut and hindgut of house fly larvae.

### Statistical analysis

All results were statistically analyzed using GraphPad Prism and Excel. The effects of different treatments on body weights and body lengths of house fly larvae were compared using two-way analysis of variance (ANOVA). Significance analysis was performed by Sidak’s multiple comparisons test (*P* < 0.05). The phenoloxidase activity in the larval hemolymph was analyzed by Student’s *t*-test. *P < 0.05, **P < 0.01, ***P < 0.001, ****P < 0.0001.

## Results

### Determination of the bacterial growth rate of *E. hormaechei* EhX and *E. hormaechei* EhY in aerobic and anaerobic states

**Fig. 1:**
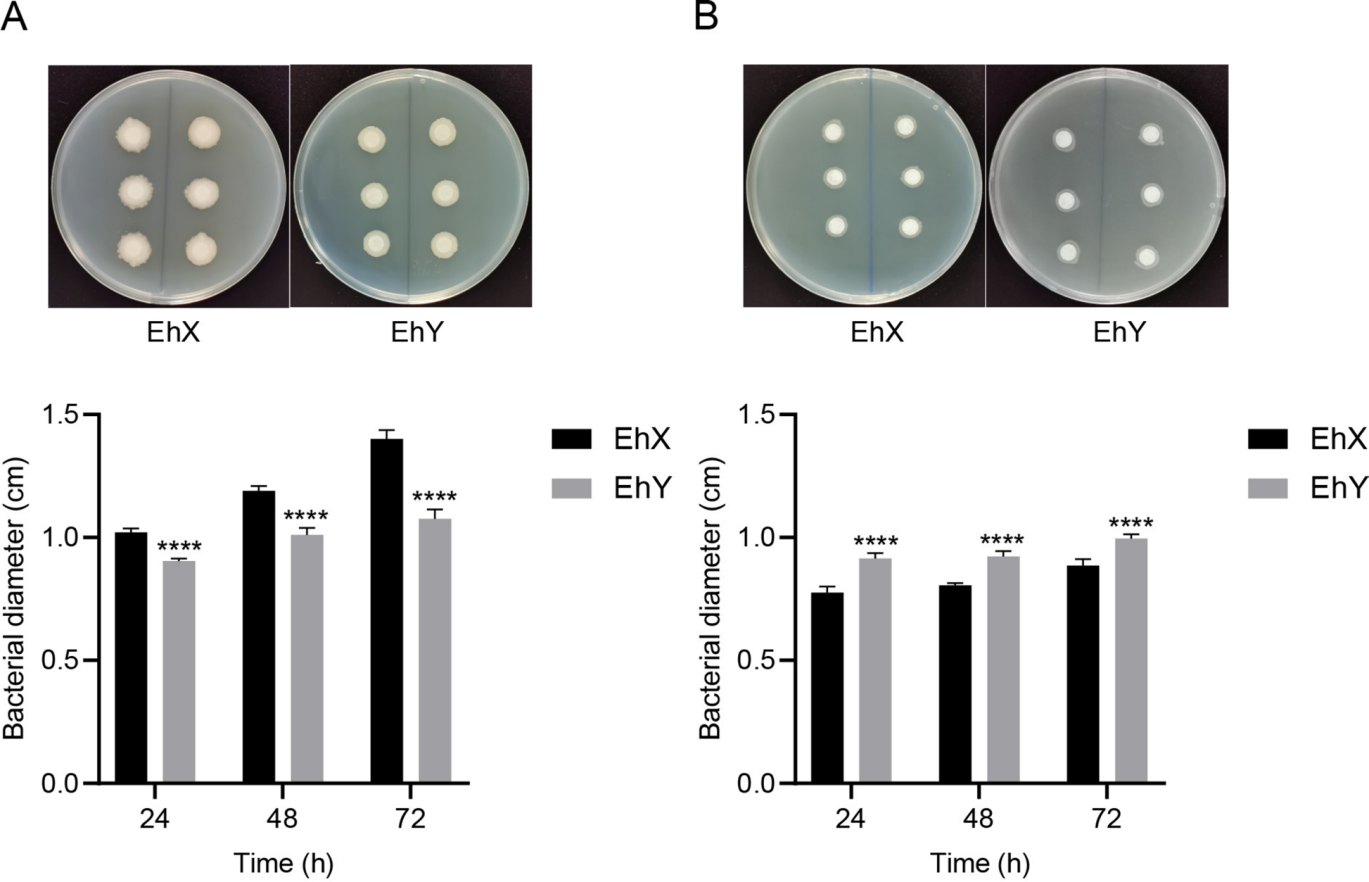
The bacterial growth rate determination of *E. hormaechei* EhX and *E. hormaechei* EhY in aerobic (**A**) and anaerobic (**B**) states. (**A**) The larger diameter of *E. hormaechei* EhX showed that it grew faster after 24, 48, and 72 h under aerobic conditions. (**B**) The larger diameter of *E. hormaechei* EhY showed that it grew faster after 24, 48, and 72 h under anaerobic conditions. An ANOVA followed by Sidak correction was used for multiple comparisons. *P < 0.05, **P < 0.01, ***P < 0.001, ****P < 0.0001

Owing to the distinct aerobic and anaerobic characteristics of *E. hormaechei* EhX and *E. hormaechei* EhY, their growth varied when cultured in differing oxygen environments, leading to variations in colony sizes on solid culture media. As determined by bacterial growth rate, larger diameters of *E. hormaechei* EhX and *E. hormaechei* EhY revealed faster growth after 24, 48, and 72 h under aerobic and anaerobic conditions, respectively. This suggests that *E. hormaechei* EhX and *E. hormaechei* EhY play major roles in aerobic and anaerobic states, respectively (Fig. 1).

### Isolation of phages from *E. hormaechei*

**Fig. 2:**
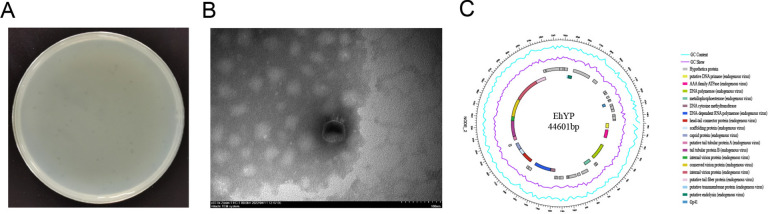
Morphology and annotated genome maps of phage EhYP. (**A**) Morphology of phage EhYP plaques in nutrient agar medium. The plaques of EhYP are medium in size and transparent. (**B**) Electron micrograph of the negatively stained, purified phage EhYP used in this study. (**C**) Annotated genome maps for the phage EhYP. In the circular genome map, the outermost black circle represents the full length of the genome, the innermost multicolored circle represents annotated functional proteins, the second outermost blue circle represents GC skew, the third outermost purple circle represents GC content, and the fourth outermost grey circle represents hypothetical proteins. EhYP, phage EhYP of *Enterobacter hormaechei* EhY

**Fig. 3:**
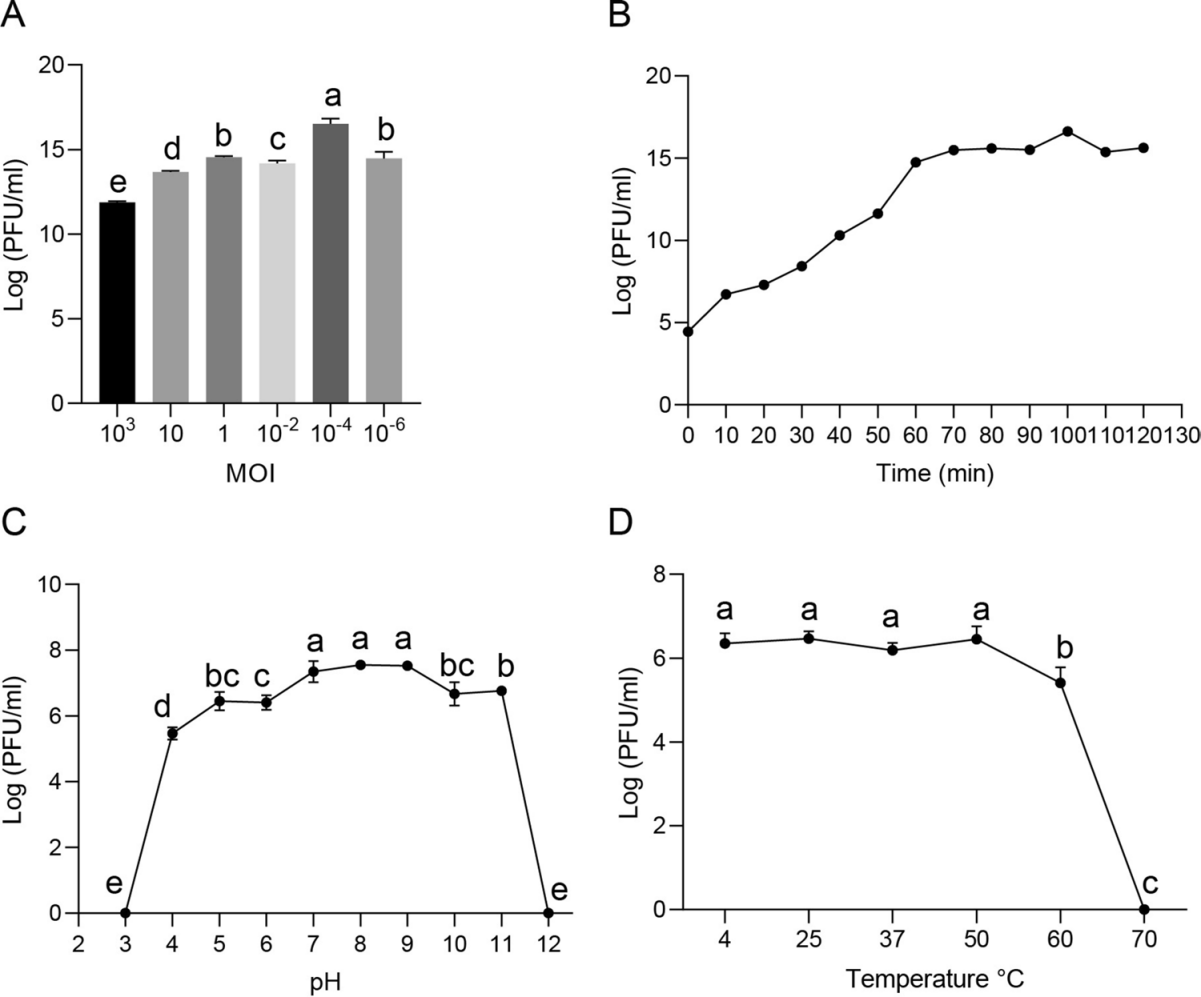
Analysis of the biological characteristics of phage EhYP. (**A**) The MOI of phage EhYP. The *E. hormaechei* EhY strain was infected with phage EhYP at various MOIs (10^3^, 10, 1, 10^− 2^, 10^− 4^, and 10^− 6^) and incubated at 37 °C for 3.5 h. When the MOI was 10^− 4^, the titer reached the highest value (16.52 log PFU mL^−1^). (**B**) One-step growth curve of phage EhYP. Phage EhYP has a latent period of 20 min. The phage titer increased rapidly and reached the highest value in 100 min. The titer of the sample was measured at different time points. (**C**) The pH stability of phage EhYP. Phage EhYP is stable over a relatively wide range of pH values, and it (∼10^8^ PFU mL^−1^) was incubated in PBS at different pH values ranging from 2 to 12 at 37 °C for 1 h. (**D**) The thermal stability of phage EhYP determined at 4, 25, 37, 50, 60, and 70 °C for 1 h, and is stable from 4 °C to 60 °C for 1 h. The data were compared by one-way ANOVA. The Brown–Forsythe test was used for significance analysis. Values are the means ± standard deviations from triplicates of each treatment

The EhYP phage of *E. hormaechei* EhY was isolated from sewage and produced a uniform plaque on the double agar plate (Fig. 2A). The EhXP phage of *E. hormaechei* EhX was isolated in our previous research [33]. The phage EhXP exhibited a favorable dissolution effect on *E. hormaechei* EhX while the phage EhYP exhibited a good lytic effect on *E. hormaechei* EhY. Furthermore, the host range of these two phages is relatively narrow, and thus cannot infect other nontargeted bacteria isolated from the larval gut (Table S1). In our previous study, the head of the EhXP phage, belonging to the *Drexlerviridae* family, has an icosahedral structure with an unretractable tail [33]. The head of the phage EhYP had a typical icosahedral structure and a short tail, which was consistent with the characteristics of the *Autographicviridae* family (Fig. 2B). The one-step growth curve showed that the latency phase of the phage EhYP was approximately 20 min and the burst size was 4.22 × 10^16^/2.95 × 10^4^ = 1.43 × 10^12^ plaque forming units (PFU) cell^−1^ (Fig. 3B). When the MOI (multiplicity of infection) was 10^− 4^, the phage EhYP titer reached the highest level, approximately 16.52 log PFU ml^−1^, indicating that the optimal multiplicity of infection (OMOI) of phage EhYP was 10^− 4^ (Fig. 3a). The phage EhYP shows excellent viability, withstands pH 5–11 for 1 h, and temperatures from 4 °C to 60 °C for 1 h (Fig. 3C, D). The biological characteristics of the EhXP phage have been studied in our previous research [33]. The genome sequencing analysis showed the total length of the EhXP genome is 52,494 bp [33], and the length of the EhYP genome is 44,601 bp (Fig. 2C).

### Effects of *E. hormaechei* on the growth and development of house fly larvae

**Fig. 4:**
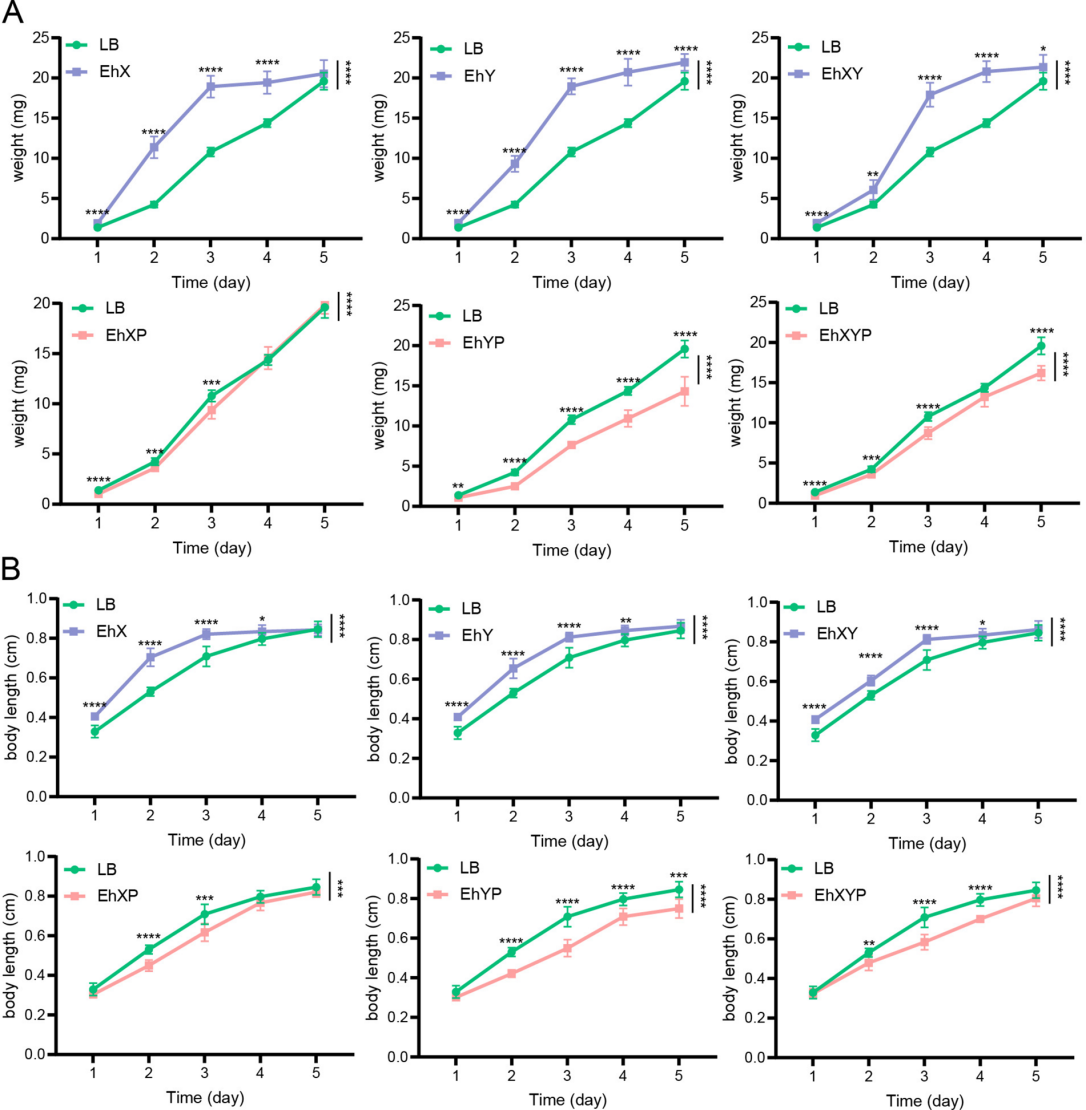
Effects of different treatments on the growth and development of house fly larvae. Different treatments had significant effects on the body weight (**A**) and body length (**B**) of house fly larvae. LB, EhX, EhY, EhXY, EhXP, EhYP, and EhXYP were cultured in Luria Bertani (LB) liquid medium, and *E. hormaechei* EhX (10^9^ CFU mL^−1^), *E. hormaechei* EhY (10^9^ CFU mL^−1^), *E. hormaechei* EhX (10^9^ CFU mL^−1^) and *E. hormaechei* EhY (10^9^ CFU mL^−1^), *E. hormaechei* phage EhXP (10^7^ PFU mL^−1^), *E. hormaechei* phage EhYP (10^7^ PFU mL^−1^), and *E. hormaechei* phage EhXP (10^7^ PFU mL^−1^) and *E. hormaechei* phage EhYP (10^7^ PFU mL^−1^) were fed to house fly larvae. Each treatment included twelve biological repeats. The data are expressed as the means ± SEMs. Repeated measures ANOVA followed by Sidak correction was used for multiple comparisons. *P < 0.05, **P < 0.01, ***P < 0.001, ****P < 0.0001

Larva development was significantly promoted in all bacterial treatment groups, but there was no synergistic effect of feeding a mixture of *E. hormaechei* EhX and *E. hormaechei* EhY. The development of all the house flies fed with bacteriophages were inhibited, especially in the EhYP group, which showed a more significant inhibitory effect. However, inhibition of the mixed feeding of phage EhXP and phage EhYP had no obvious synergistic effect (Fig. 4). These results indicate that the addition of *E. hormaechei* has a positive impact on larval development, and phage-mediated knockdown of *E. hormaechei* has a negative impact, threatening larval health.

### Effects of the addition and removal of *E. hormaechei* on the crawling ability of house fly larvae

Digitizer^®^ 4 was used to label crawling traces on media after 15 min (Fig. S1). The crawling trace experiment showed that larvae had weaker motility, slow crawling, and a significantly reduced number of crawling traces in all phage-treated groups. There was no significant difference in the motility of house fly larvae in all bacteria-treated groups.

### Effects of *E. hormaechei* addition and removal on the gut microbiome of house fly larvae

We further assessed the dynamic composition and diversity of the gut bacteria in the various treatment groups. The 16S rRNA genes were sequenced in the gut bacteria of the house fly larvae of the LB, EhX, EhY, EhXY, EhXP, EhXP, EhYP, and EhXYP groups. A total of 2,946,596 high-quality bacterial sequences were obtained from all samples, with sequence numbers 116479–163124. These sequences were normalized and aggregated into 4455 OTUs with 97% similarity between all samples. The rarefaction curves revealed that the depth of the sequencing data was sufficient for the data to represent the majority of species diversity and abundance (Fig. S2).

**Fig. 5:**
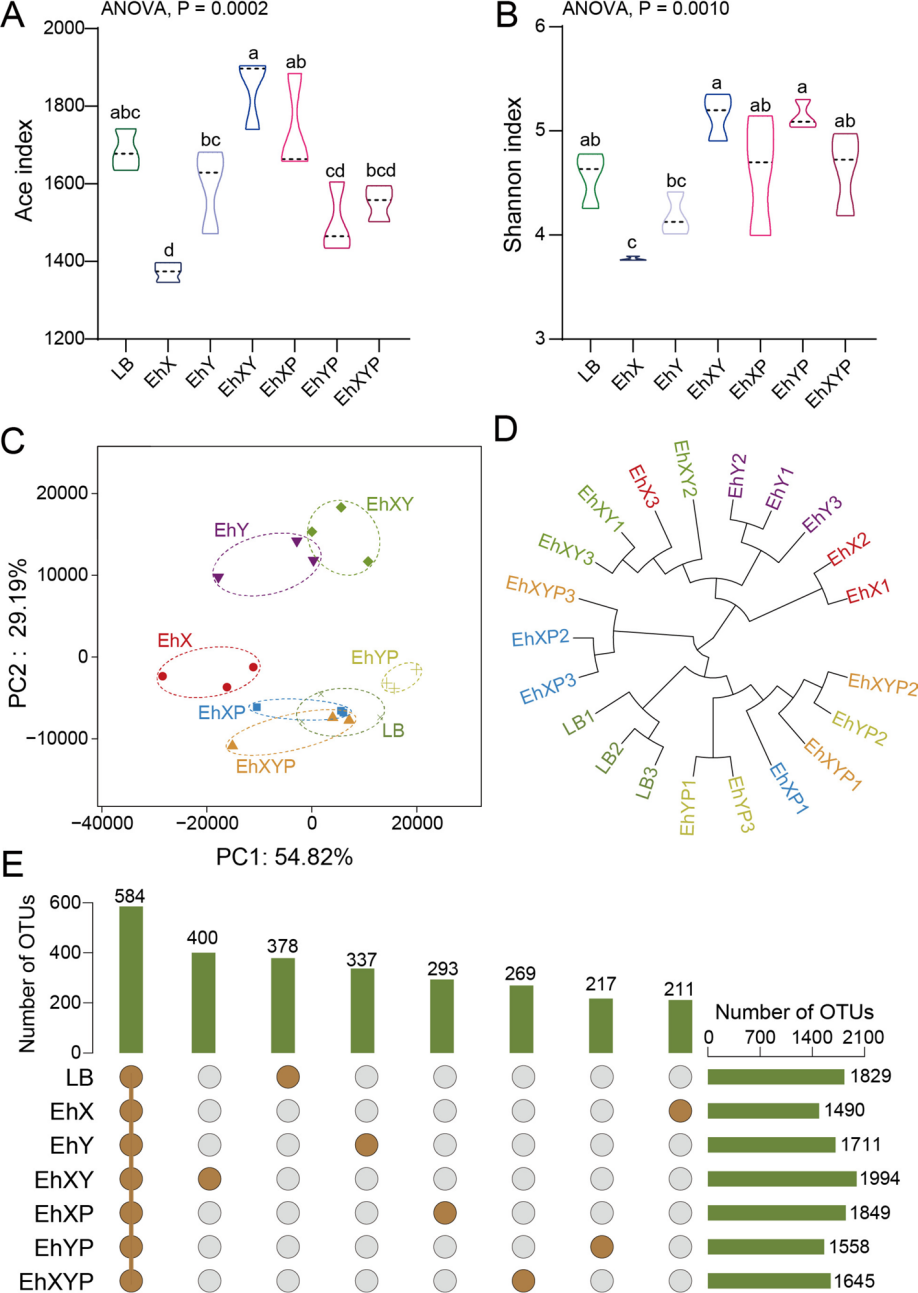
Bacterial richness and diversity of samples. (**A**) Chao1 index. (**B**) Shannon index. The data were compared by one-way ANOVA. The Brown–Forsythe test was used for significance analysis. Values are the means ± standard deviations from triplicates of each treatment. (**C**) Principal coordinate analysis (PCoA) of bacterial community structure in seven groups. Each symbol represents a sample of gut bacteria. (**D**) Unweighted pair-group method with arithmetic mean (UPGMA) tree analysis of the samples. (**E**) Venn diagram analysis of unique and shared OTUs of the gut bacteria in house fly larval samples. The number represents the number of unique OTUs in each sample and common OTUs shared by two or more samples

The addition or lack of *E. hormaechei* (EhX, EhY) significantly interfered with the larval gut microbiome (Fig. 5). Ace and Simpson indices indicated bacterial diversity was altered in the different treatment groups (Fig. 5A, B). The principal coordinate analysis (PCoA) analysis revealed gathering of the phage-treated and bacteria-treated groups together, indicating significant differences in microbial communities between them (Fig. 5C). In addition, compared with *E. hormaechei* EhX, the removal of *E. hormaechei* EhY had a more significant impact on the structure of the house fly gut microbiome, which is consistent with their effects on the growth and development of house flies.

**Fig. 6:**
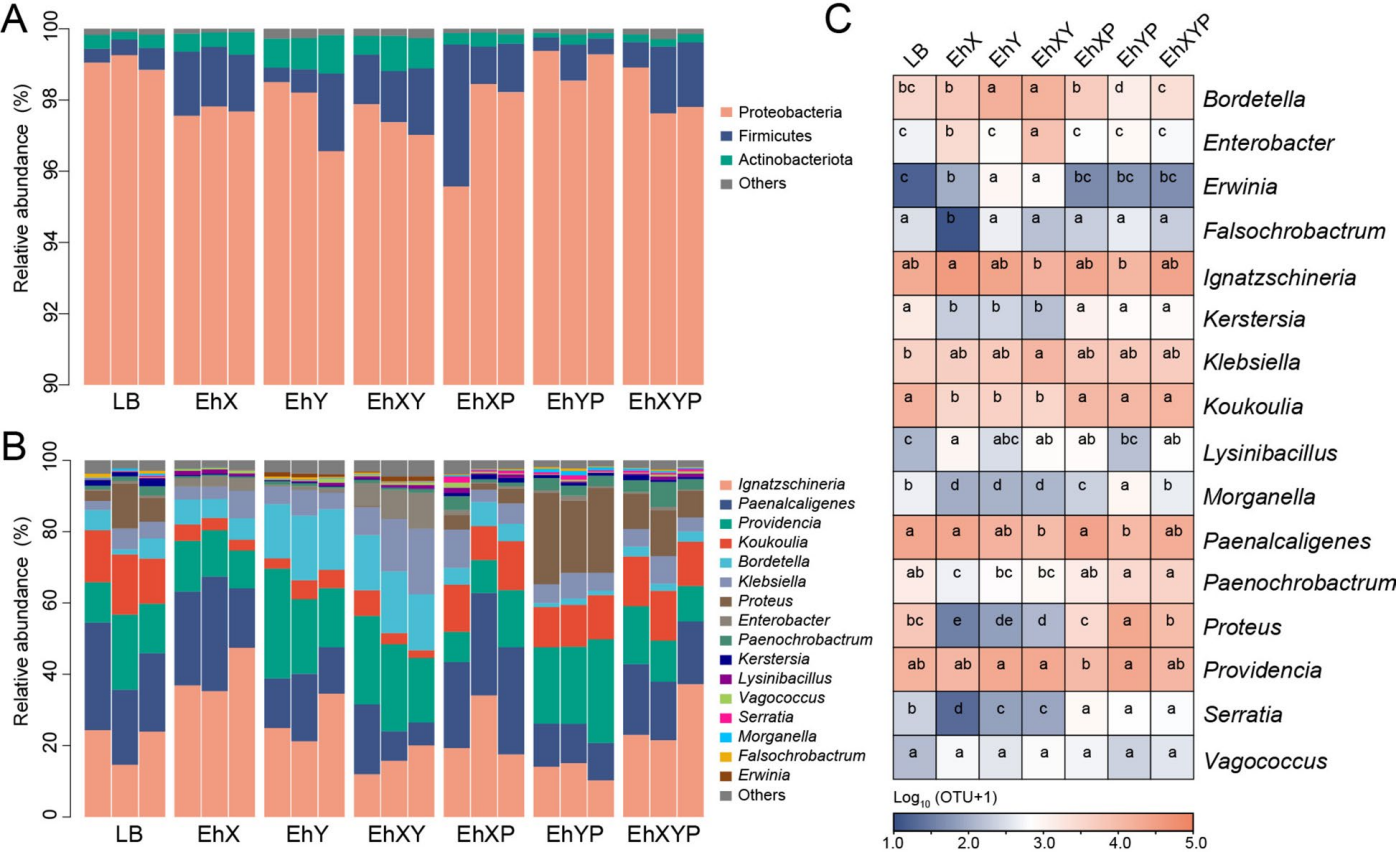
The relative abundances of the top three phyla and the top 16 genera of gut bacteria in different treatment groups. (**A**) Relative abundances of the top three phyla in house fly larval samples. (**B**) The relative abundances and distributions of the top 16 genera in house fly larval samples. (**C**) Dynamic variation in the OTU number of key bacteria in different groups. The data were compared by one-way ANOVA. The Brown–Forsythe test was used for significance analysis. The different letters in the row indicate statistically significant differences (*P* < 0.05)

House fly larvae from different treatment groups exhibited slight differences at the phylum level, while there were different bacterial community structures at the genus level (Fig. 6). Among the 16 most abundant genera in house fly larval gut, beneficial bacteria, such as *Klebsiella*, significantly increased in abundance in the bacterial treatment groups (EhX, EhY, and EhXY), while harmful bacteria, such as *Serratia* and *Morganella*, decreased significantly (Fig. 6). In addition, beneficial bacteria such as *Enterobacter* also increased significantly in abundance in the bacterial treatment groups (EhX and EhXY) (Fig. 6). *Serratia* increased significantly in abundance in the EhXP, EhYP, and EhXYP phage treatment groups, while *Providencia* and *Morganella* increased in the EhYP phage treatment group (Fig. 6). Therefore, the composition of the intestinal bacterial community of house fly larvae was greatly altered in the bacteria-treated groups. Although phages exhibit host specificity, the cascade effect on other nontargeted bacterial species affects the composition of the gut bacterial community of house fly larvae.

Antagonism assays were carried out to determine the interaction between culturable bacteria and *E. hormaechei* (EhX/EhY). The assays revealed that growth of *E. hormaechei* EhX, *P. aeruginosa* Y12*, P. stuartii* Ps, and *P. vermicola* Pv was inhibited by high abundances of *E. hormaechei* EhY (Fig. S3). The antagonism assay in a previous study revealed that growth of *P. aeruginosa* Y12*, P. stuartii* Ps, and *P. vermicola* Pv was inhibited by a high abundance of *E. hormaechei* EhX [33]. Furthermore, *E. hormaechei* EhY was inhibited by high abundances of *E. hormaechei* EhX (Fig. S3).

### Network of gut microbiome in larvae treated with different diets

**Fig. 7:**
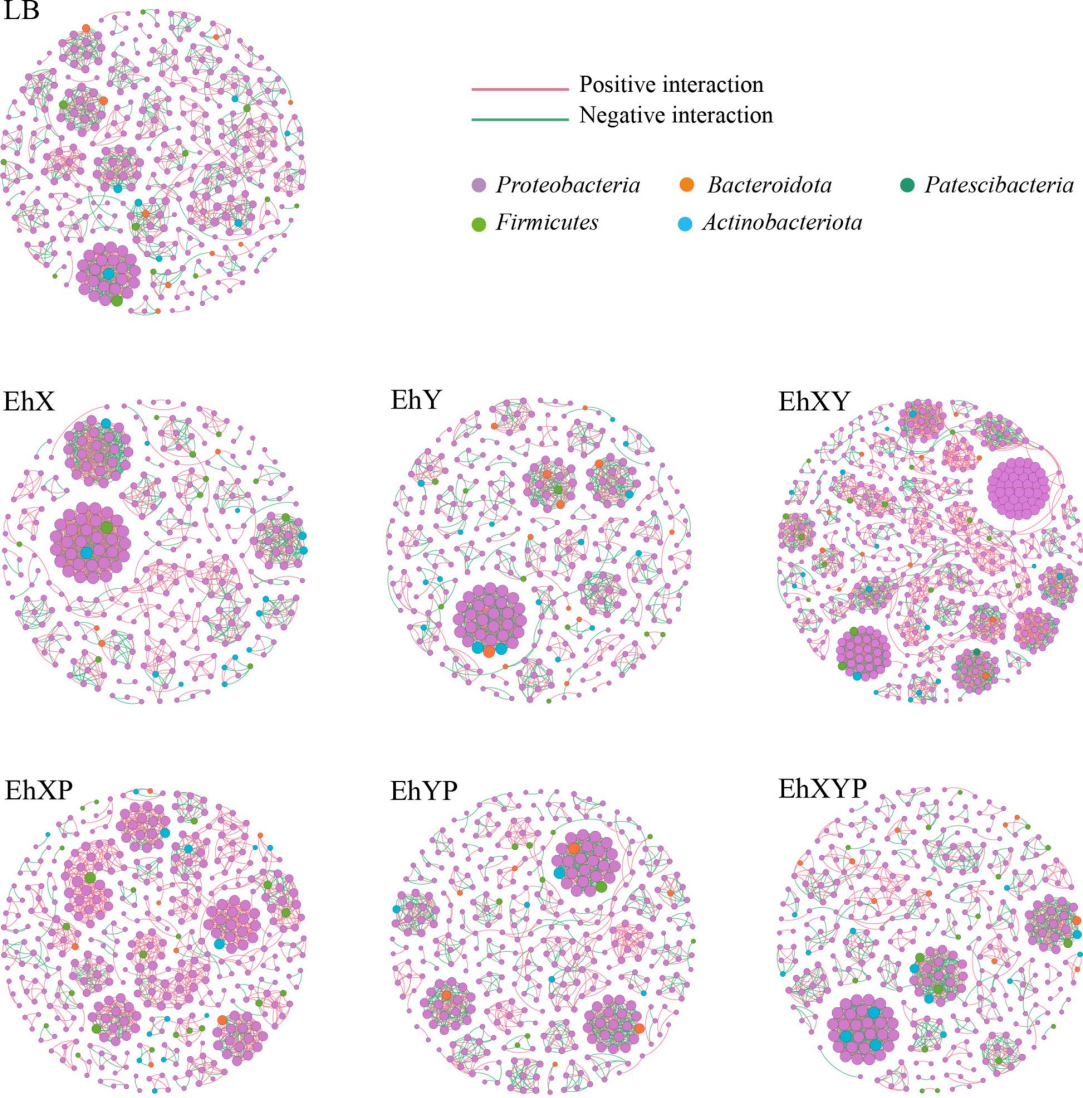
Gut bacterial cooccurrence microbiome networks between different processing groups. Each point in the graph represents a species, and those related species are connected by a line. The red lines represent positive correlations, and the green lines represent negative correlations. The node colors represent the taxon classification at the phylum level

To analyze bacterial correlations within the bacterial community, a network was constructed based on Spearman’s correlations. In all samples, a high degree of connectivity between *Proteobacteria* was observed (Fig. 7). However, compared with the control group (92.84% in LB), the interaction between *Proteobacteria* decreased in the bacterial treatment group (91.36% in EhX, 89.64% in EhY, and 92.6% in EhXY) and the phage-treated groups treated with phage (90.07% in EhXP and 89.41% in EhXYP), while the interaction between *Proteobacteria* increased in the group (94.33% in EhYP). We speculate that there is a decrease in the association between some harmful bacteria, such as *Serratia* and *Morganella*, and *Proteobacteria* in the bacterial treatment groups (EhX, EhY, and EhXY). Beneficial bacteria, for example *Enterobacter* increased in the bacterial treatment group (EhX, EhXY). In the phage-treated group (EhXP and EhXYP), the decrease in some beneficial bacteria increased the correlation between harmful bacteria and *Proteobacteria* e.g., *Serratia*. In the phage-treated group (EhYP), there was an increased correlation between some harmful bacteria and *Proteobacteria*, such as *Serratia* and *Providence*. Based on these results, alteration of the loads of specific bacteria in the gut could greatly affect the bacterial interactions among the microbial communities.

### Effects of the addition and removal of *E. hormaechei* on phenoloxidase activity in house fly larvae

**Fig. 8:**
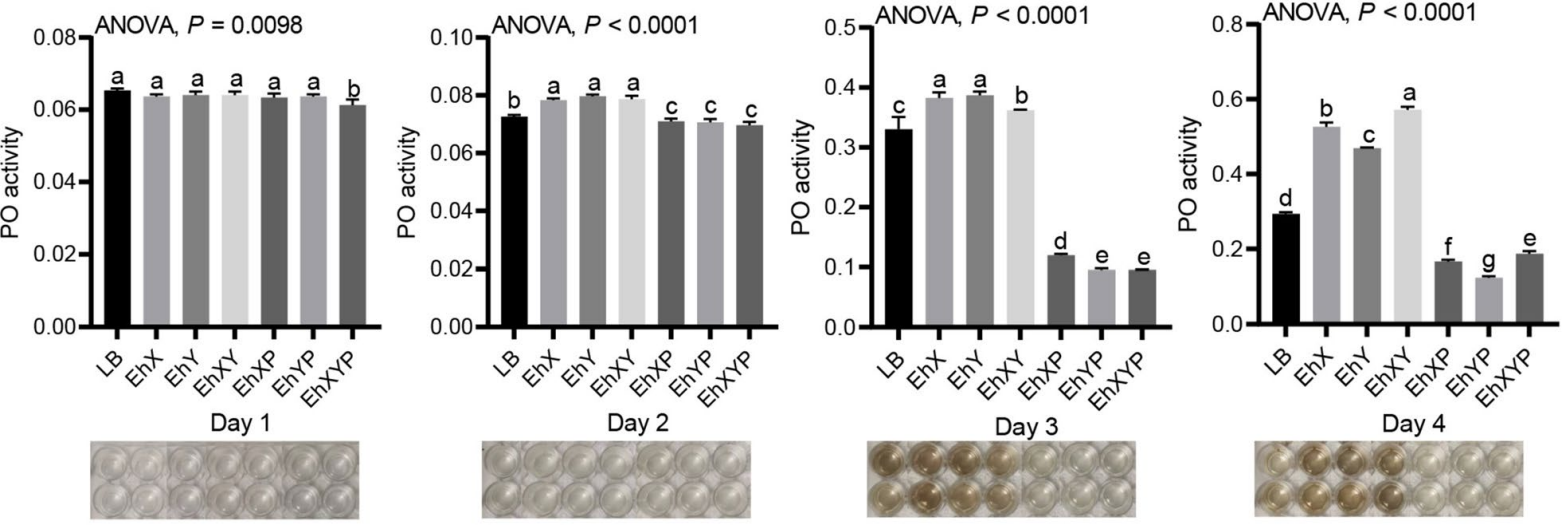
Effects of different treatments on phenoloxidase activity in house fly larvae. LB, EhX, EhY, EhXY, EhXP, EhYP, and EhXYP were cultured in Luria Bertani (LB) liquid medium, and *E. hormaechei* EhX (10^9^ CFU mL^−1^), *E. hormaechei* EhY (10^9^ CFU mL^−1^), *E. hormaechei* EhX (10^9^ CFU mL^−1^) and *E. hormaechei* EhY (10^9^ CFU mL^−1^), phage EhXP (10^7^ PFU mL^−1^), phage EhYP (10^7^ PFU mL^−1^), and phage EhXP (10^7^ PFU mL^−1^) and phage EhYP (10^7^ PFU mL^−1^) were fed to house fly larvae. The data were compared by one-way ANOVA. The Brown–Forsythe test was used for significance analysis. Values are the means ± standard deviations from triplicates of each treatment

To study the immune response by which house flies resist the invasion of pathogenic bacteria, the effects of feeding *E. hormaechei* and phages on phenoloxidase activity in the larval hemolymph were analyzed. Larval phenoloxidase activity increased significantly in all bacteria-treated groups (Fig. 8), while it decreased in all phage-treated groups, over 2, 3, and 4 days (Fig. 8).

### Bacterial abundance assay of *E. hormaechei* EhX/EhY in house fly larvae under different conditions

**Fig. 9:**
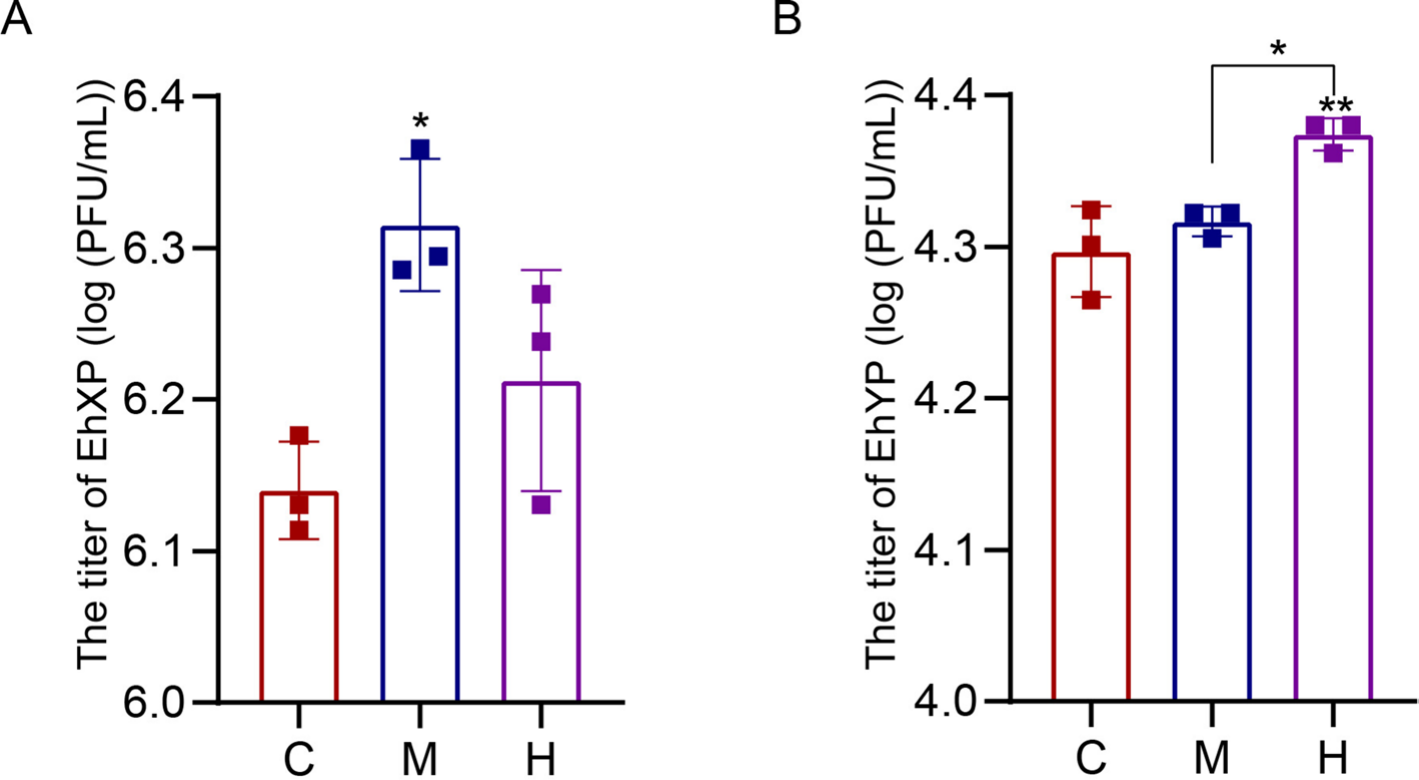
Bacterial assay of *Enterobacter hormaechei* EhX/EhY under different conditions. (**A**) The titers of EhXP in different treatment groups from the midgut and hindgut of house fly larvae. (**B**) The titers of EhYP in different groups from the midgut and hindgut of house fly larvae. C, control group; M, midgut group; H, hindgut group. The data were compared by one-way ANOVA. The Brown–Forsythe test was used for significance analysis. Values are the means ± standard deviations from triplicates of each treatment

The midgut and hindgut maintain different oxygen concentrations in house fly larvae. To analyze the dynamic alterations of *E. hormaechei* EhX/EhY in the gut of house fly larvae at different oxygen concentrations, the bacterial load of *E. hormaechei* EhX/EhY was tested in the midgut and hindgut of house fly larvae. As the phage EhXP/EhYP rapidly amplifies only in the presence of its host bacteria *E. hormaechei* EhX/EhY, the titers of the phages EhXP/EhYP revealed higher bacterial load of aerobic *E. hormaechei* EhX and anaerobic *E. hormaechei* EhY in the high-oxygen midgut and low-oxygen hindgut, respectively (Fig. 9). Our results revealed aerobic *E. hormaechei* EhX played an important role in the aerobic midgut, while facultative anaerobic *E. hormaechei* EhY played an important role in the anaerobic hindgut (Fig. 9). Aerobic/facultative anaerobic *E. hormaechei* EhX/EhY establishes competition and cooperation, forming a negative correlation “pedal effect” to maintain the constant composition of gut bacteria in house fly larvae.

## Discussion

In this study, larval development and phenoloxidase activity in larval hemolymph increased in all the bacteria-treated groups, and decreased in all the phage-treated groups. Furthermore, the addition of *E. hormaechei* EhX/EhY or phage EhXP/EhYP altered the gut microbiome, which we speculate significantly impacts house fly larvae. These results suggest that phage predation of susceptible bacteria causes cascading effects on other microbial species, leading to changes in gut microbiome structure that can affect host health and potentially cause disease.

Pathogenic bacteria can negatively impact larval growth and even cause death [23], but the role of beneficial bacteria in larval development requires further study. This research investigated the effects of *E. hormaechei* EhX/EhY and phage EhXP/EhYP on house fly larvae. The addition of *E. hormaechei* EhX/EhY promoted the growth of beneficial bacteria, inhibited harmful bacteria, and enhanced larval development. Conversely, phage EhXP/EhYP reduced the bacterial abundance of *E. hormaechei* EhX/EhY, altered the gut microbiome composition, and promoted the growth of harmful bacteria, thereby inhibiting larval development.

Among the 16 genera most abundant in the gut microbiome of house fly larvae, *Klebsiella* was significantly increased in the bacterial treatment groups (EhX, EhY, and EhXY), while *Serratia* and *Morganella* showed a downward trend. Furthermore, *Enterobacter* and *Klebsiella* also increased significantly in the bacterial treatment groups (EhX and EhXY). The abundance of *Enterobacter* in house fly larvae fed high concentrations of *E. hormaechei* increased significantly in a short period, which may be related to the higher concentration of *E. hormaechei* in the larval diet. Our previous research showed that *E. hormaechei* accelerated larval growth by inhibiting the growth of some pathogenic strains and increasing the bacterial load of beneficial flora to modulate the gut microbiome [45]. The addition of *Enterobacter* sp. to the diet can improve the recovery rate of egg–pupa and egg–adult, and shorten the development time of egg–pupa, pupa, and egg–adult stages of *Cerfly capitata* [46]. *Enterobacter* sp. AA26 gut symbiont as a protein source is considered an important component of the insect diet, which benefits Mediterranean fruit fly mass-rearing and sterile insect technique applications [47]. The application of *Enterobacter* sp. probiotics improved the productivity of the Mediterranean fruit fly (medfly), as well as reduced rearing duration [48]. The germ-free Red palm weevil larvae fed *Enterobacter cloacae* increased their hemolymph triglyceride and glucose content markedly, which affects the development of this pest by regulating its nutrition metabolism [49]. Diverse members of *Enterobacteriaceae*, most commonly *Klebsiella* and *Enterobacter* bacteria are found in the guts of fruit flies, which play a crucial role in host nutrition, development, physiology, and resistance to insecticides and pathogens [50]. Multiresistance *Klebsiella pneumoniae* such as BSFL7-B-5 (from the middle midgut of 7-day BSFL) and BSFL11-C-1 (from the posterior midgut of 11-day BSFL) in the gut of black soldier fly larvae (BSFL) helped the larvae against sulfonamides (SAs) or cadmium (Cd) stress. Given these roles of *Enterobacteriaceae*, we believe that *Klebsiella* and *Enterobacter* could play an active role in their early development. The increased bacterial abundance of *E. hormaechei* EhX/EhY can affect the composition of the gut microbiome, promote the growth of beneficial bacteria, and thus promote larval development.

A decrease in the abundance of *Serratia* and *Morganella* was observed in the larval gut after feeding *E. hormaechei* EhX/EhY. Pathogens, such as *Morganella morganii* and *Serratia marcescens,*, showed higher abundances in multiple-mated females, supporting the hypothesis that promiscuity turned over the abundance of beneficial and detrimental microbiomes in *Spodoptera frugiperda* females [51]. *M. morganii* 3A2A were highly toxic to *Galleria mellonella* larvae, suggesting the presence of potential virulent factors in this strain [52]. *S. marcescens* facilitated arboviral infection by secreting SmEnhancin protein, which digests membrane-bound mucins in mosquito gut epithelia [53]. Mortality was slightly higher for *Tenebrio molitor* larvae fed on *Serratia marcescens*-inoculated wheat bran [54]. *Serratia marcescens* VA is highly virulent to mosquito larvae in a prodigiosin-dependent manner [55]. Therefore, we believe that the increase in the bacterial abundance of *E. hormaechei* EhX/EhY resulted in the reduction of some harmful bacteria in the larval gut, which further favored the proliferation of other beneficial bacteria in the house fly larval gut and facilitated the development of the house fly larvae.

To analyze the interactions between *E. hormaechei* and other culturable bacteria, a plate antagonism assay was performed. There was obvious antagonism between the two strains of *E. hormaechei* and “harmful bacteria” such as *P. aeruginosa* Y12*, P. stuartii* Ps, and *P. vermicola* Pv in the gut of house fly larvae. In addition, the two bacteria also compete. Therefore, feeding *E. hormaechei* in large quantities can promote larval development by inhibiting some harmful bacteria. There is competition between *E. hormaechei* EhX and *E. hormaechei* EhY. The proliferation of the *E. hormaechei* EhX strain will affect the proliferation of the other *E. hormaechei* EhY strains. Therefore, mixed feeding of the two strains has no synergistic effect on larval development.

Larval growth was significantly inhibited by the treatment of single phage EhXP or EhYP, but the inhibition of the combined treatment of the two phages did not have a synergistic effect, indicating that *E. hormaechei* EhX and *E. hormaechei* EhY competition and cooperation were inhibited in the larval gut. After a certain decrease in *E. hormaechei* EhX, mediated by the phage EhXP, *E. hormaechei* EhY tended to increase, because the inhibitory effect of *E. hormaechei* EhX on *E. hormaechei* EhY was weakened. Unlike the upward trend of *E. hormaechei* EhY, the downward trend of *E. hormaechei* EhY was mediated by the phage EhYP. Furthermore, while *E. hormaechei* EhX decreased, *E. hormaechei* EhY relatively increased, and the two bacterial population levels reached a certain dynamic balance. In addition, *E. hormaechei* EhX exists in a hyperoxic environment, while *E. hormaechei* EhY exists in a hypoxic environment. Aerobic *E. hormaechei* EhX or facultative anaerobic *E. hormaechei* EhY had the reverse correlation “pedal effect” in regulating larval growth. Therefore, the two strains of *E. hormaechei* play a role together in different environments of oxygen content. Two strains of *E. hormaechei* compete and cooperate in the larval intestine to promote the development of the larva. The removal of *E. hormaechei* EhY, primarily present in the anaerobic hindgut, had a more significant impact on house fly development, suggesting its crucial role in nutrient reabsorption. Future research will focus on the specific function of anaerobic *E. hormaechei* EhY in the hindgut of house flies.

The insect gut microbiome is dynamic, and interactions between strains are crucial for insect health. This study examined the gut microbiome structure in house fly larvae following changes in *E. hormaechei* abundance, using phages as precise tools.

## Conclusions

This study investigated the dynamic changes in the gut microbiome caused by aerobic and facultative anaerobic strains of *E. hormaechei* using phages as a powerful tool. The removal of facultative anaerobic *E. hormaechei* EhY had a more pronounced inhibitory effect on larval growth than the aerobic *E. hormaechei* EhX, while the addition of both strains promoted development. Further analysis revealed that aerobic *E. hormaechei* EhX and facultative anaerobic *E. hormaechei* EhY, with the reverse correlation “pedal effect,” play an important role in the aerobic midgut and anaerobic hindgut of the house fly, respectively. Our work reveals the important contribution of gut aerobic/anaerobic microbes in house fly larvae and provides future insights to elucidate the interplay between phage, gut aerobic/anaerobic microbes, and insect health.

## Supplementary Information

Additional file 1.

## Data Availability

Phage sequences were deposited in the NCBI (EhXP: Phc, MZ669808, EhYP: OQ884031). The 16s sequence data of the microbiome were stored in the SRA database (BioProject accession number: PRJNA909407).

## Abbreviations

16S rRNA: 16S ribosomal RNA
OTU: Operational taxonomic unit
