# Supplementary material for: Pedal effect: aerobic and facultative anaerobic Enterobacter hormaechei maintain the homeostasis of the house fly gut microbiota under different oxygen conditions
Source: Parasit Vectors. 2025 Dec 6;19:32. doi: 10.1186/s13071-025-07013-5 (PMC12817646; doi:10.1186/s13071-025-07013-5)
Supplement: Supplementary file 1 — Additional file 1. [file 13071_2025_7013_MOESM1_ESM.docx]

**Supplementary materials**

**Supplementary Table:**

**Table S1** **Infectivity range of phages against intestinal bacteria of house fly larvae.** Phage EhYP were spotted onto lawns of each bacteria and incubated aerobically overnight at 37 °C. Zones of clearing indicated infectivity. (+) = lysis; (-) = no lysis

| Bacteria | Phage |
| --- | --- |
|  | EhYP |
| *E.hormaechei* EhY | ＋ |
| *E.hormaechei* EhX | － |
| *Klebsiella pneumoniae* KX | － |
| *Pseudomonas aeruginosa* Y12 | － |
| *Acinetobacter bereziniae* Ab | － |
| *Providencia stuartii* Ps | － |
| *E. cloacae* Ec | － |
| *Lactococcus lactis* Ll | － |
| *Lysinibacillus fusiformis* Lf | － |
| *P. Vermicola* Pv | － |
| *Bacillus safensis* Bs | － |

**Supplementary Figure:**

**
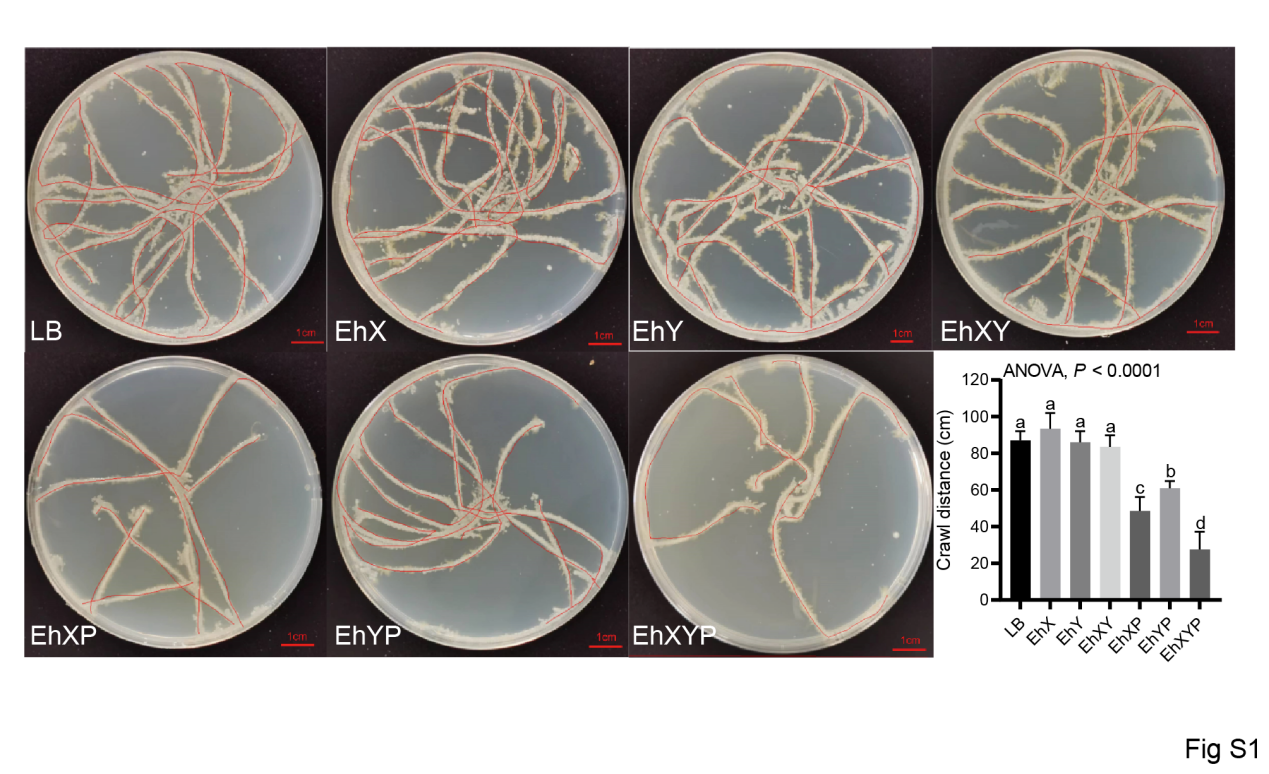
Fig S1. Effects of different treatments on crawling ability of house fly larvae.** Different treatments had significant effects on the crawling trail and crawling distance of house fly larvae. LB, EhX, EhY, EhXY, EhXP, EhYP and EhXYP were cultured in Luria Bertani (LB) liquid medium, and *E. hormaechei* EhX (10^9^ CFU/mL), *E. hormaechei* EhY (10^9^ CFU/mL), *E. hormaechei* EhX (10^9^ CFU/mL) and E. hormaechei EhY (10^9^ CFU/mL), phage EhXP (10^7^ PFU/mL), phage EhYP (10^7^ PFU/mL), phage EhXP (10^7^ PFU/mL) and phage EhYP (10^7^ PFU/mL) were fed to house fly larvae. Each treatment included ten biological repeats. The data were compared by one-way ANOVA. The Brown-Forsythe test was used for significance analysis. Values are the means ± standard deviations from triplicates of each treatment.


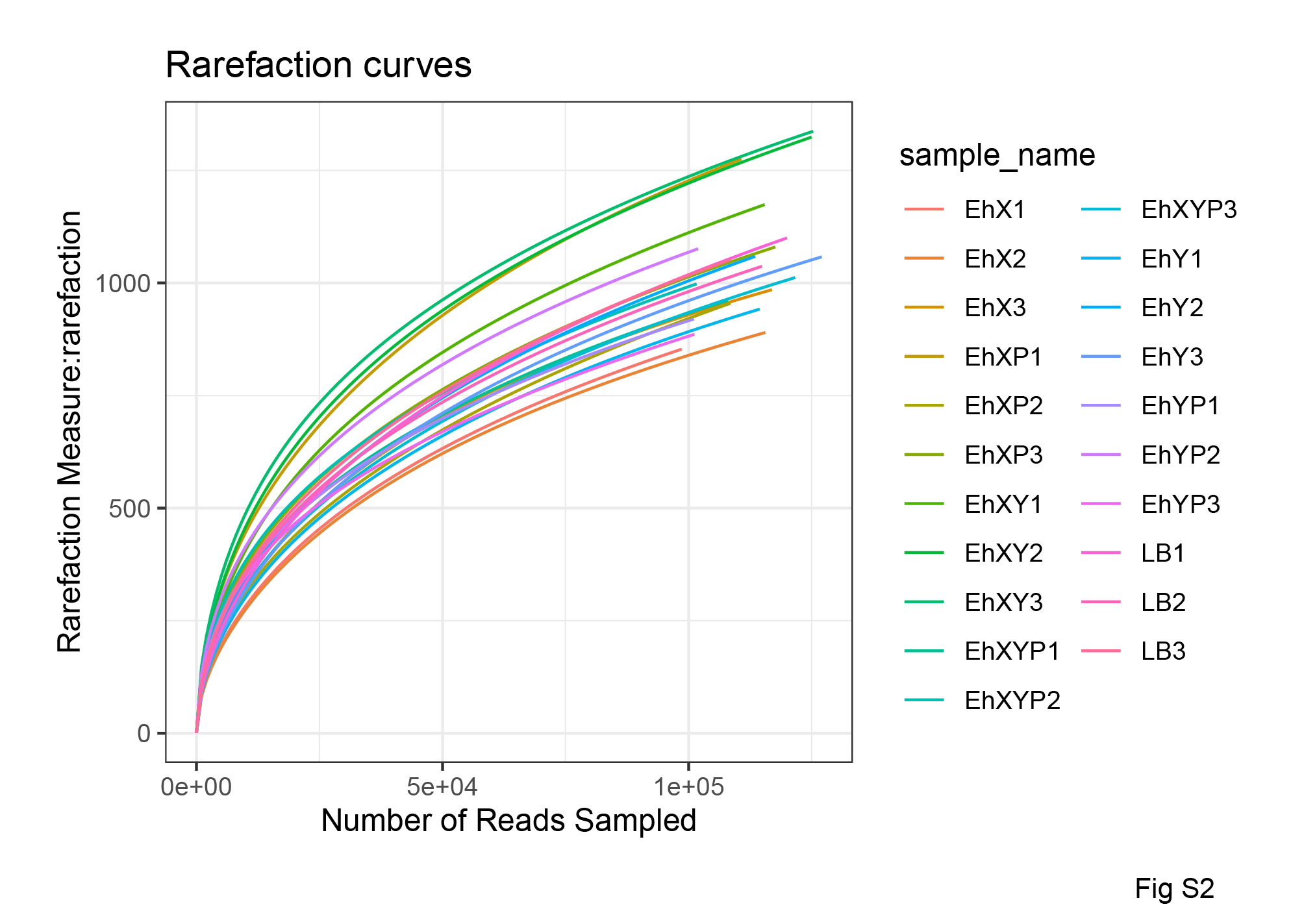


**Fig S2.** **Species rarefaction analysis of sample sequences among different groups.** Rarefaction curves showing the relationship between sequences per sample and Operational Taxonomic Unit (OTU) resolution.


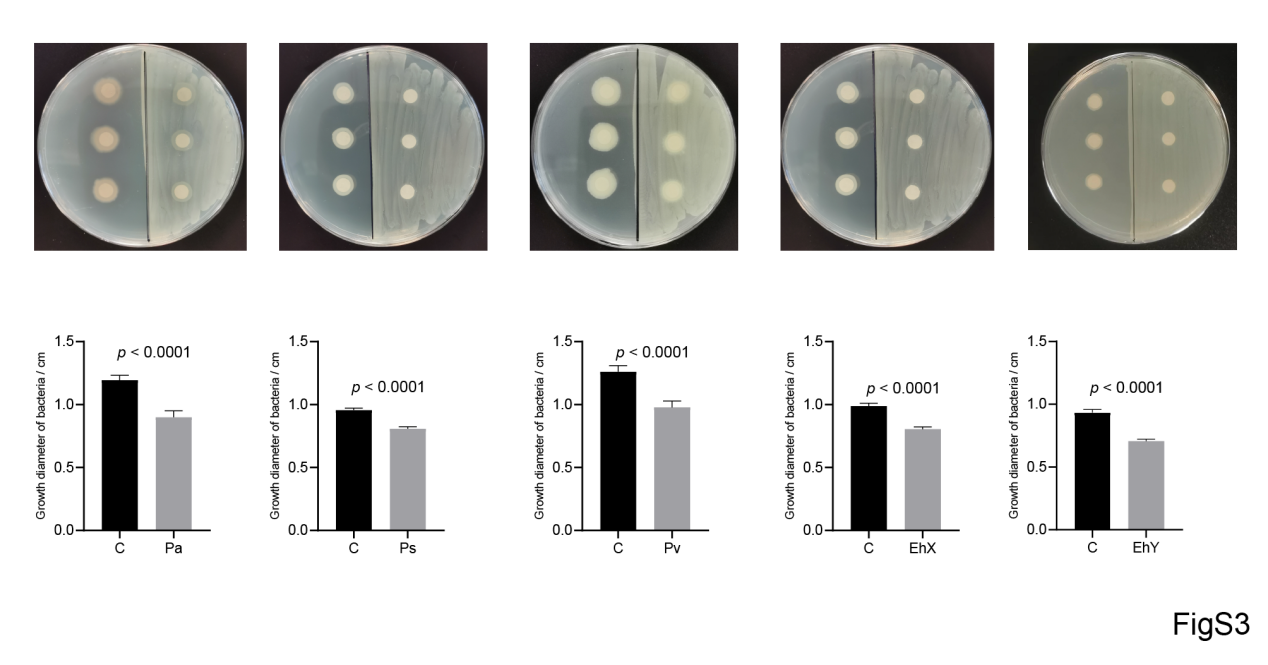


**Fig S3.** **Antagonism experiment comparing *E. hormaechei* EhY and cultivable bacteria in the house fly larval gut.** Antagonism experiment comparing *E. hormaechei* EhY and cultivable bacteria, including *Pseudomonas aeruginosa* Y12*, Providencia stuartii* Ps*, Providencia vermicola* Pv and *E. hormaechei* EhX in the first four pictures. Pa: *P. aeruginosa* Y12; Ps: *P. stuartii* Ps; Pv: *P. vermicola* Pv; EhX: *E. hormaechei* EhX. The last panel shows the antagonism experiment between *E. hormaechei* EhX and *E. hormaechei* EhY. Data are shown as the means ± SEMs. The t test was used for the statistical analysis.
